# Near-infrared prediction of tannin content in walnut kernels using wavelet transform combined with interpretable machine learning models

**DOI:** 10.3389/fpls.2026.1746869

**Published:** 2026-02-06

**Authors:** Qiuhao Xia, Langqin Luo, Yerhazi Yerzati, Mian Muhammad Ahmed, Yonghao Chen, Shiwei Wang, Jiangnan Qin, Liping Chen, Qiang Jin, Zhongzhong Guo, Rui Zhang

**Affiliations:** 1 College of Horticulture and Forestry, Tarim University, Alar, China; 2 Efficient and High-Quality Cultivation and Deep Processing Technology of Characteristic Fruit Trees in Southern Xinjiang, National Local Joint Engineering Laboratory, Alar, China; 3 Xinjiang Production and Construction Corps, Southern Xinjiang Characteristic Forest and Fruit Technology Innovation Center, Alar, China; 4 College of Life Science and Technology, Tarim University, Alar, China; 5 Beijing Academy of Agricultural and Forestry Sciences Forestry Fruit Tree Research Institute, Beijing, China; 6 School of Forestry and Landscape Architecture, Xinjiang Agricultural University, Urumqi, China; 7 School of Information Engineering, Tarim University, Aral, China

**Keywords:** continuous wavelet transform (CWT), near-infrared, random forest (RF), Shapley additive explanations (SHAP), tannins

## Abstract

**Introduction:**

Tannin content is a key factor influencing the taste of walnuts and serves as an important index for evaluating walnut quality. Rapid and accurate detection of tannin levels in walnut kernels is therefore significant for quality assessment and management. This study aims to develop an efficient method for predicting tannin content in walnut kernels using near-infrared (NIR) spectroscopy combined with machine learning techniques.

**Methods:**

A total of 180 samples of ‘Wen 185’ walnut kernels were used as the research objects. The NIR reflectance spectra of the samples were measured within the range of 4000–10000 cm⁻¹. The spectral data were processed using mathematical transformations and continuous wavelet transform (CWT), both separately and in combination. Pearson correlation analysis was applied to extract characteristic bands related to tannin content. Based on these features, a random forest (RF) model was constructed to quantitatively predict tannin content. Additionally, the SHAP algorithm was employed to interpret and visualize the machine learning model.

**Results:**

The results indicated that within the spectral range of 4000–10000 cm⁻¹, the NIR reflectance of walnut kernels increased with tannin content under different orchard management modes. Both first-order differential transformation and CWT, as well as their combination, significantly enhanced the correlation between spectral data and tannin content. The combination of first-order differential transformation and CWT notably improved the model's prediction performance. The optimal prediction model was achieved using the feature lg’(1/R)_CWT_28, with training set metrics of R² = 0.880, RMSE = 1.188, RPD = 2.904, and validation set metrics of R² = 0.831, RMSE = 1.620, RPD = 2.459.

**Discussion:**

The study demonstrates that combining mathematical transformations with wavelet transform can effectively improve the prediction accuracy of models for tannin content in walnut kernels. The RF model based on processed spectral data showed strong performance, indicating its potential for rapid and non-destructive tannin quantification. The use of SHAP algorithm further enhances model interpretability. These findings provide a valuable reference for the accurate prediction of tannin content in walnut kernels and may support quality control in walnut production and processing.

## 1 Introduction

Walnut *(Juglans* regia L.) is a significant woody oil and economic tree species in China, valued for its drought tolerance and the high nutritional quality of its fruit. In particular, the southern Xinjiang region has seen extensive cultivation of walnuts, which have become a crucial component of local economic development and a primary source of income for farmers (Pei and Lu, 2011; Qu, 1980). Currently, however, most walnut products in China are either consumed directly or subjected to minimal processing. The astringency of the fruit notably affects its taste, with tannin compounds being the principal contributors to this astringency (Liu et al., 2023). Walnut tannin is a highly polymerized polyphenolic compound that interacts with salivary proteins, resulting in astringency. Research indicates that walnut tannin possesses antioxidant and antibacterial properties and may also play a role in the prevention of chronic diseases (Xiao et al., 2006; Aoki et al., 2006; Li et al., 2009).

Traditional methods for detecting tannins in walnuts typically include EDTA titration, the phosphomolybdic acid-sodium tungstate colorimetric method, capillary gas chromatography, and potassium ferrous hexachloride (III) spectrophotometry (Huang and Ni, 2022; Cai, 1997; Hernes and Hedges, 2000; Yang and Qu, 1989). However, these techniques are often costly, exhibit low time efficiency, and pose challenges for large-scale rapid detection. Furthermore, the use of chemical reagents during testing can endanger the health of testers, while the disposal of chemical waste can contribute to environmental pollution (Ma et al., 2022).

With the rapid advancement of spectroscopic technologies, near-infrared (NIR) spectroscopy has evolved from a well-established diagnostic method into a continuously innovating tool with expanding applications and significant practical value. NIR spectroscopy enables real-time, non-destructive, and dynamic monitoring of crop quality at specific spatial and temporal scales, offering considerable advantages over traditional chemical or sensory methods. As such, developing a rapid, universal, and efficient approach to predict tannin content in Juglans regia ‘Wen 185’ walnuts grown in southern Xinjiang has become increasingly important for rapid quality assessment and classification. Zhang et al. (2011) established a calibration model for soluble tannin content in astringent persimmons using visible and near-infrared diffuse reflectance (Vis/NIR) spectroscopy. By applying an improved partial least squares regression (PLSR) algorithm combined with first derivative and scatter correction preprocessing, the model demonstrated superior predictive performance, highlighting the utility of Vis/NIR spectroscopy for internal quality assessment. Similarly, Cheng (2020) employed NIR hyperspectral imaging integrated with chemometric methods, machine learning, and deep learning techniques to rapidly classify wine grape varieties, determine their geographic origins, and predict tannin levels at different maturation stages. Jensen et al. (2008) utilized Fourier transform mid-infrared (FT-MIR) spectroscopy for the rapid quantification of various wine constituents, including tannins. However, due to overlapping spectral responses from other compounds, accurate quantification of tannins remains challenging. Their study explored four variable selection methods to identify key spectral regions relevant to tannin determination using PLSR. In another study, Ying et al. (2006) applied wavelet transform (WT) to denoise NIR spectra of 90 apple samples, exploiting the multiscale differences in the evolution of wavelet modulus maxima between singular signals and random noise, and successfully predicted sugar content via stepwise regression.NIR spectroscopy has thus been widely applied in predicting tannin content in crops such as grapes, apples, persimmons, and sorghum. However, limited studies have specifically addressed tannin quantification in walnut kernels. Existing research has predominantly focused on optimizing model performance, while relatively little attention has been given to enhancing model interpretability.

In this experiment, spectral information from walnut kernels was collected within the wavenumber range of 4000–10000 cm^-1^.Various spectral processing methods, including mathematical transformation, wavelet transformation, and their combinations, were investigated to identify the most suitable pretreatment method for detecting tannin content in walnut kernels. Building on this foundation, a prediction model for tannin content was developed using random forests. The SHAP algorithm was applied to ascertain feature significance and facilitate internal model visualization, enabling swift tannin content detection in walnut kernels.

## 2 Materials and methods

### 2.1 Plant materials and instrumentation

The experimental material for this study was the ‘Wen 185’ walnut selected from the walnut forest farm in Wensu County, Aksu Prefecture, Xinjiang. A total of 180 walnut samples were collected from 9 walnut orchards with varying management levels: 3 high-yield, 3 medium-yield, and 3 low-yield orchards. The walnut trees in these orchards are spaced 5 meters by 6 meters apart and are all 10 years old. Following harvest at ripeness, the walnuts underwent a process where the green skins were removed, and then they were dried in a well-ventilated environment until their moisture content reached approximately 6%. Subsequently, the walnuts were shelled, kernels extracted, crushed for 3 minutes using a FW-80 high-speed universal crusher, and thoroughly mixed. The crushed walnut kernels were then sealed in plastic bags and stored at 4 °C for subsequent spectral scanning and determination of tannin content.

### 2.2 Acquisition and processing of raw spectral data from walnut kernels

NIR spectral data were collected using a Fourier-transform near-infrared spectrometer (Antaris II, Thermo Fisher Scientific, USA). The instrument was operated at a resolution of 8 cm^-1^ with a gain setting of 2, using the built-in background as reference. Each spectrum was obtained as the average of 32 scans. Prior to spectral acquisition, walnut samples were equilibrated under controlled environmental conditions (25°C and 40% relative humidity) for 24 hours to ensure consistency with the instrument’s ambient environment, thereby minimizing spectral variability.

The instrument was preheated for 60 minutes before measurement. Spectra were recorded over the wavenumber range of 4000-10000 cm^-1^. Ground walnut kernel powder was uniformly packed into quartz sample cups (30 mm diameter, 5 mm height, 1 mm wall thickness), with the sample surface leveled and aligned with the rim of the cup. Each sample was scanned three times, resulting in a total of 540 spectra for 180 samples. The final representative spectrum for each sample was obtained by averaging its three replicate scans. After each measurement, the sample cups were sequentially rinsed with tap water, distilled water, and then wiped clean with ethanol to ensure cleanliness and prevent cross-contamination.

#### 2.2.1 Outlier detection and removal

Outlier removal was performed using the Monte Carlo method (Meng et al., 2022), which is effective for identifying data points that deviate significantly from the distribution of the dataset. Such outliers may result from instrumental noise, measurement errors, or data entry mistakes. Eliminating these anomalous points is essential to enhance the accuracy and robustness of subsequent model development.

#### 2.2.2 Correlation analysis

Selection of informative spectral features is a critical step in improving the sensitivity of NIR data to tannin content. In the preliminary phase of this study, various feature selection strategies were evaluated, including Pearson correlation analysis. The comparison indicated that spectral bands selected based on a significance threshold of p <0.01 yielded superior modeling performance. Consequently, Pearson correlation analysis was conducted using MATLAB R2023a (MathWorks, USA) to assess the strength of association between each spectral band and tannin content. Spectral bands exhibiting statistically significant correlations (p<0.01) were retained for model construction (Mao et al., 2023). The Pearson correlation coefficient (r), ranging from -1 to 1, quantifies the degree of linear association between variables, with larger absolute values indicating stronger correlations.

#### 2.2.3 Traditional mathematical transformations

To evaluate the impact of different preprocessing methods on spectral feature extraction and avoid information omission, this study used a total of 11 mathematical transformations, including reciprocal transformation, logarithmic transformation, and their derivatives, for horizontal comparison. Among them, reciprocal transformation aims to compress high reflection areas to enhance low value signals, while logarithmic transformation is used to reduce dynamic range and improve spectral response linearity. Both are commonly used benchmark methods to verify the effectiveness of spectral preprocessing.

#### 2.2.4 Wavelet transform processing

Continuous Wavelet Transform (CWT) is a time-frequency analytical method that decomposes spectral reflectance into components of different frequencies and scales, allowing the identification of subtle spectral variations across multiple resolutions. This study employed various mother wavelets—including Bior, Morlet, Haar, and Gabor functions (Li X. et al., 2024), to convolve with the spectral data, thereby generating wavelet coefficients corresponding to different scales and frequency domains (Guan et al., 2024). The multiscale decomposition enabled by CWT improves feature resolution by capturing localized changes in spectral patterns while suppressing random noise, ultimately enhancing data interpretability and model performance (Lin et al., 2021). The wavelet decomposition is mathematically expressed as:

(1) $$\omega_{ij}=\int_{-\infty }^{+\infty }v_{ij}\psi_{a,b}(j)dj$$

In the formula, they represent the wavelet coefficient and reflectance of the j-th band of the i-th tannin sample, respectively; *a* is the scale factor ranging from 2^1^ to 2^10^, *b* is the translation factor; *ψa,b(j)* denotes the wavelet basis function.

(2) $$\psi_{a,b}(j)=\frac{1}{\sqrt{a}}\psi \left(\frac{j-b}{a}\right)$$

In addition, CWT encompasses a variety of wavelet basis functions, each of which may yield different decomposition outcomes. The selection of an appropriate wavelet function and optimal decomposition scale is therefore critical for effective spectral preprocessing.To select the optimal wavelet basis function, this study preliminarily compared various commonly used wavelets, including Daubechies (db4), Symlets (sym8), Morlet (morl), Mexican hat (mexh), and Gaussian function (gaus4). Based on the comprehensive performance of feature band separation and noise suppression in pre experiments, the gaus4 wavelet was ultimately selected for subsequent analysis (Li et al., 2024).

### 2.3 Determination of tannin content in walnut kernels

Tannin content was determined according to the Chinese agricultural industry standard NY/T 1600-2008: Determination of tannin content in fruits, vegetables, and their products—Spectrophotometric method (Li et al., 1999). Precisely 1.00 g of ground walnut kernel sample was weighed and placed in a 100 mL volumetric flask. The sample was extracted using a boiling water bath for 30 minutes. After extraction, the mixture was cooled to room temperature and diluted to the mark with distilled water. The extract was centrifuged, and 2 mL of the supernatant was transferred into a 50 mL volumetric flask. Then, 1 mL of a sodium tungstate–sodium molybdate reagent and sodium carbonate solution was added, and the mixture was shaken thoroughly. After standing at room temperature for 2 hours, the absorbance of the solution was measured at 765 nm using a UV–Vis spectrophotometer.The tannin content was calculated using the following equation:

(3) $$X_{1}=\frac{C\times V_{1}\times N}{M\times 1000}$$

In the formula, X1 represents the tannin content in the sample (mg/g); *C* represents the gallic acid content obtained from the standard curve (mg); *V1* represents the volume of the sample determination solution (ml); *M* represents the mass of the walnut sample taken (g); *N* represents the dilution factor; 1000 represents the conversion coefficient.

### 2.4 Model construction and performance evaluation

With the rapid development of machine learning algorithms, numerous advanced modeling techniques have been applied to the prediction of fruit quality traits, often outperforming traditional statistical methods (Tan et al., 2023). Random forest (RF) (Breiman, 2001), an ensemble learning algorithm composed of multiple decision trees, exhibits robustness to multicollinearity and performs well with imbalanced or incomplete datasets (Guo et al., 2025). In this study, the dataset was randomly split into a training set and a validation set at a 6:4 ratio. Model performance was evaluated using the coefficient of determination (R^2^), root mean square error (RMSE), and relative percent deviation (RPD). A model with R^2^ approaching 1 and low RMSE indicates strong predictive capability. An RPD value between 1.4 and 2.0 suggests moderate reliability suitable for estimation, while RPD > 2.0 indicates a robust predictive model (Li et al., 2024).

### 2.5 SHAP-based feature importance analysis

**Figure 1:**
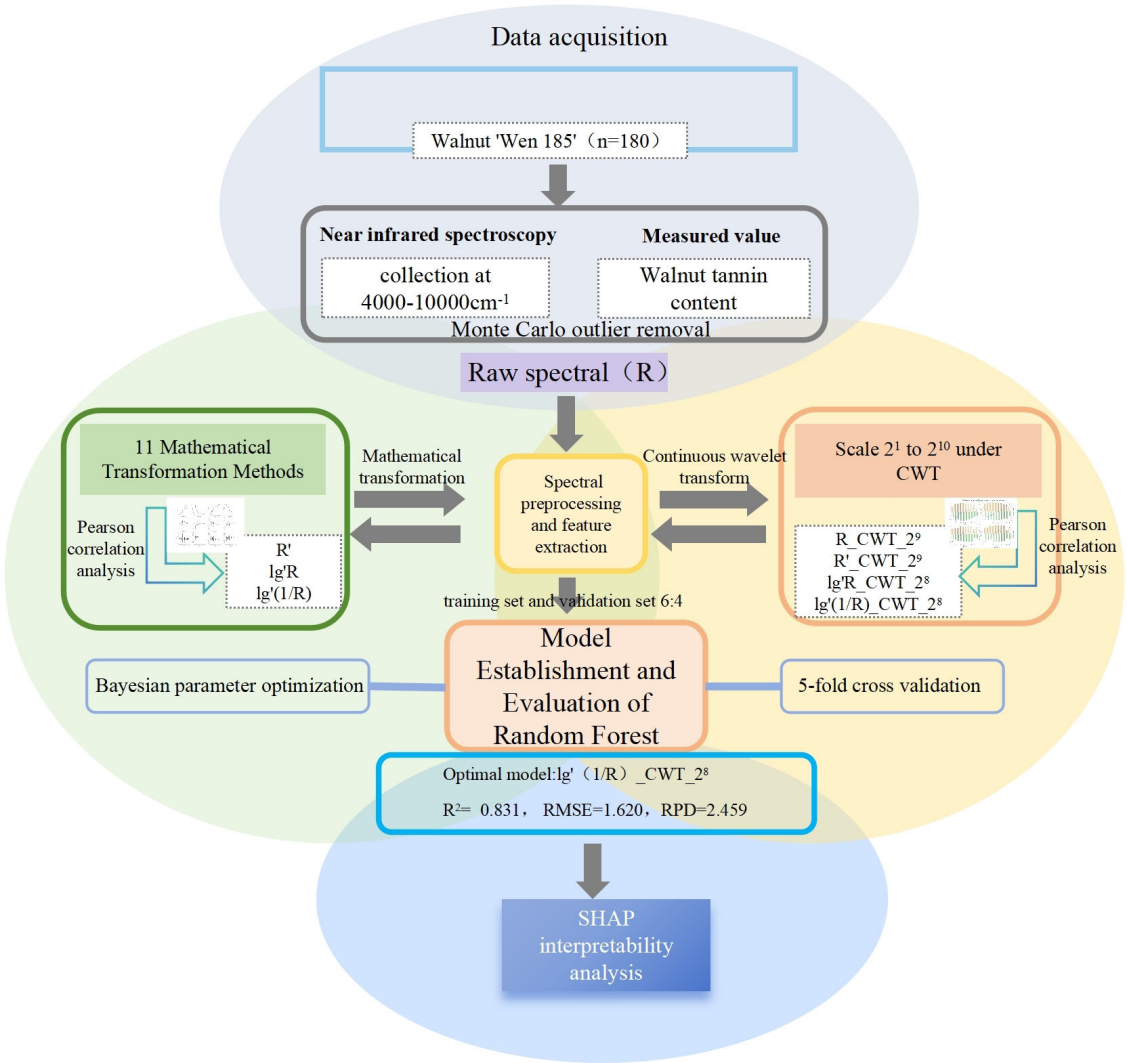
Experimental flowchart.

Due to the “black-box” nature of many machine learning models, their internal decision-making processes are often opaque and difficult to interpret (Ye et al., 2024). The SHAP (Shapley additive explanations) algorithm addresses this issue by applying Shapley values—originating from cooperative game theory-to decompose the output of a model into contributions from each input feature (Li et al., 2025). This allows for a more transparent understanding of the model’s predictions and facilitates interpretability in complex systems.The experimental flowchart is shown in **Figure 1**.

### 2.6 Software and implementation

All data preprocessing, spectral feature selection, and model development were performed using MATLAB^®^ (Version R2023a; MathWorks, 2023) and Python. Chemical and spectral mean values were calculated using Microsoft Excel^®^ (Version 2016; Microsoft Corporation, 2016). All visualizations and figures were generated using Origin^®^ (Version 2021; OriginLab Corporation, 2021). For model interpretation, the SHAP Python package (Lundberg and Lee, 2017) was employed, which is based on the Shapley additive explanations framework.

## 3 Results and analysis

### 3.1 Analysis of tannin content in walnut kernels and removal of outliers

**Figure 2:**
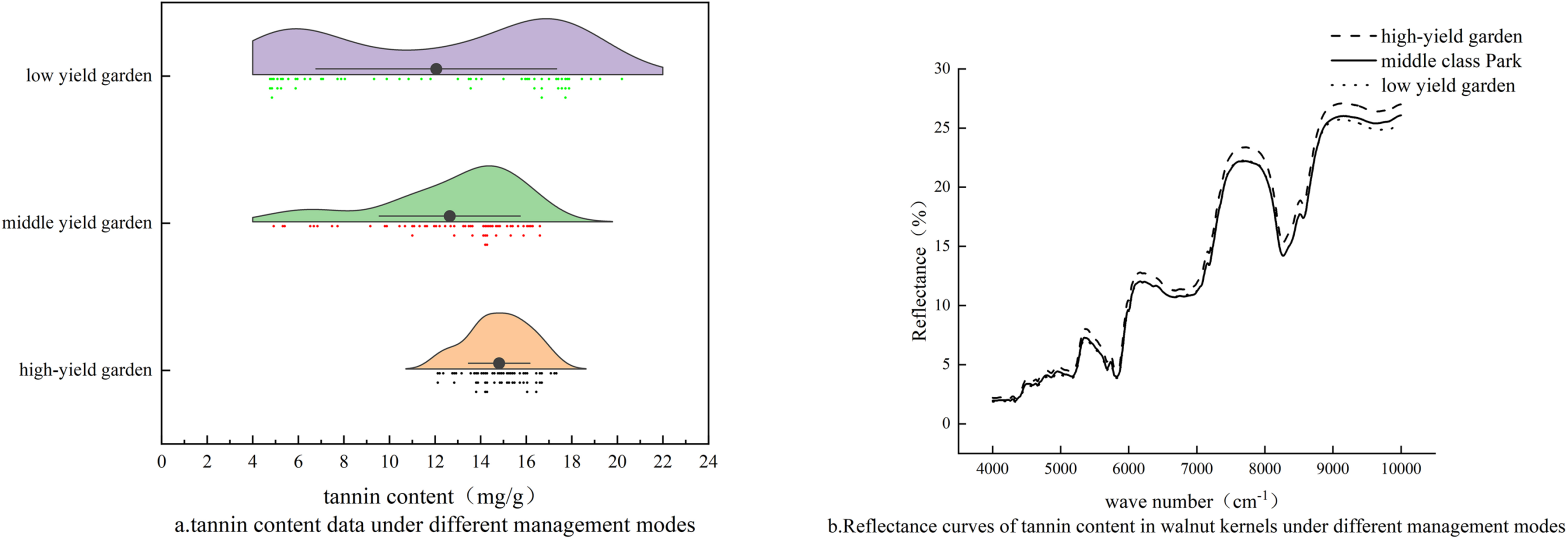
Tannin content data and reflectance curves under different management modes. **(a)** tannin content data under different management modes, **(b)** Reflectance curves of tannin content in walnut kernels under different management modes.The absorption features in the spectra, particularly around 4000–5000 cm^-1^ and 7000–9000 cm^-1^, are associated with characteristic vibrational modes of tannin molecules, primarily involving O-H overtone and combination bands.

The tannin content in walnut kernels can be determined using a specific formula (Equation 3). The results indicate significant variability in tannin content among samples, demonstrating notable distinctions and representativeness. a comparison of orchard sample data under various management models is presented in **Figure 2a**, revealing distinct differences. The average tannin content differs among orchards managed under different models, facilitating model development. **Figure 2b** illustrates the near-infrared spectral range of 4000–10000 cm^-1^. The spectral curves under different management modes exhibit similar overall shapes, running roughly parallel to each other, with spectral reflectance increasing as wavelength increases.The absorption features observed in the spectra, particularly in the regions around 4000–5000 cm^-1^ and 7000–9000 cm^-1^, are likely associated with the characteristic vibrational modes of tannin molecules. Tannins, as polyphenolic compounds, contain abundant hydroxyl (-OH) groups, whose overtone and combination bands typically appear in the NIR region. Specifically, the first overtone of O-H stretching vibrations often occurs around 7000 cm^-1^, while combination bands involving O-H bending and stretching vibrations may contribute to the absorption features in the 4000–5000 cm^-1^ range.

**Figure 3:**
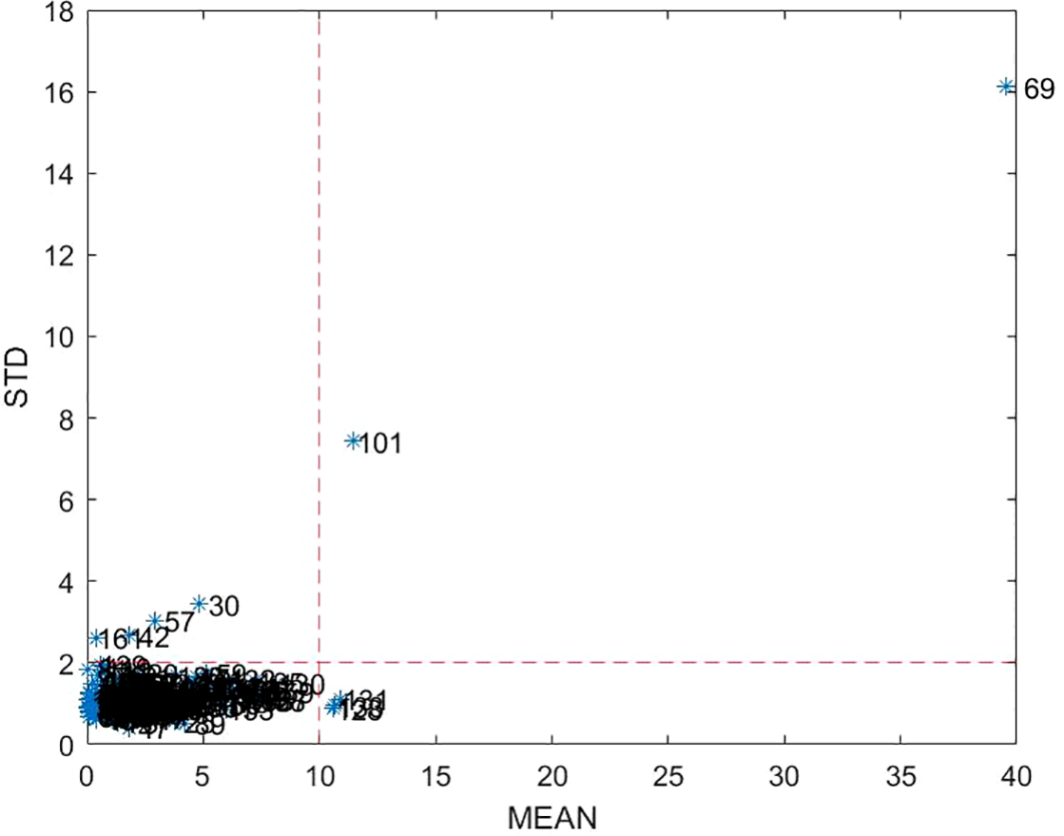
Removal of outliers in data.

Outliers in the dataset were identified and removed using the Monte Carlo simulation method. A total of 2000 iterations were performed. In each iteration, 60% of the samples were randomly selected as the training set, and the remaining 40% were used for validation. The training data were preprocessed using mean centering (“center”), and a partial least squares (PLS) regression model was constructed using 20 latent variables. The resulting regression coefficients were applied to the validation samples to obtain predicted values, and prediction errors were calculated accordingly.As shown in **Figure 3**, samples that fell outside the dashed boundary lines were identified as outliers. These samples exhibited either a mean prediction error greater than the overall mean or a standard deviation exceeding the global standard deviation. The criteria for outlier elimination were set as: standard deviation > 2 and mean > 10. Based on these thresholds, nine samples—No. 30, 42, 57, 69, 101, 128, 131, 133, and 161—were identified as outliers and removed from the dataset.After eliminating these nine samples, the remaining 171 samples were retained for subsequent modeling. This outlier removal step led to a significant improvement in model performance, enhancing the reliability and stability of the predictive results.

### 3.2 Spectral feature analysis of mathematical transformations

**Figure 4:**
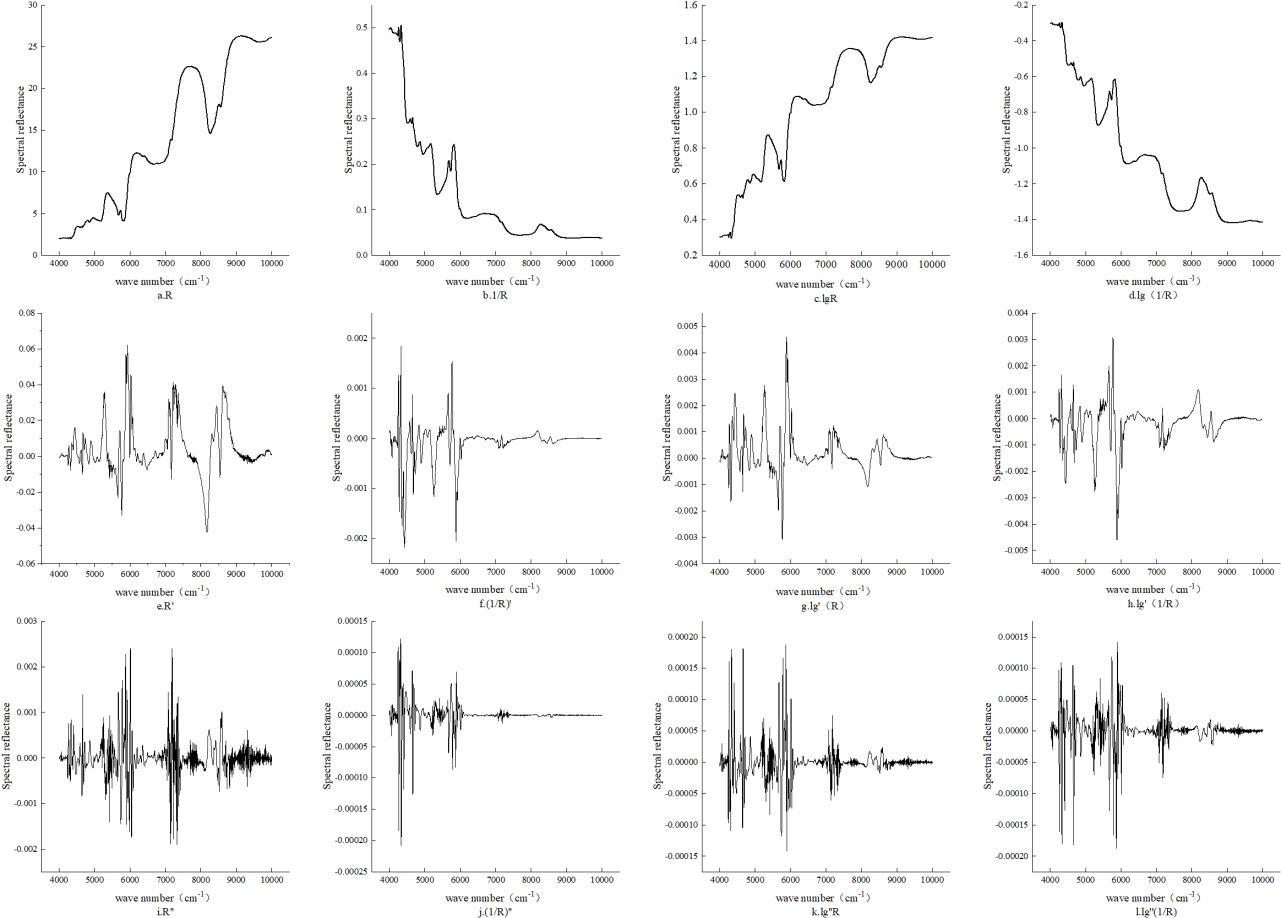
The original spectral reflectance (R) and the average reflectance of spectra processed by 11 mathematical transformations. **(a)** R; **(b)** 1/R; **(c)** lgR; **(d)** lg(1/R); **(e)** R’; **(f)** (1/R)’; **(g)** lg’R; **(h)** lg’(1/R); **(i)** R’’; **(j)** (1/R)’’; **(k)** lg’’R; **(l)** lg’’(1/R).

After applying 11 mathematical transformations to the raw reflectance spectra (R), including reciprocal (1/R), logarithmic (log R), reciprocal-logarithmic (log(1/R)), as well as first- and second-order derivatives, significant differences in spectral reflectance patterns were observed (**Figure 4**). As shown in **Figure 4a**, the original spectra are relatively smooth, lacking prominent peaks or absorption valleys, making it difficult to extract subtle features associated with walnut kernel tannin content. **Figures 4b-d** display the results of the 1/R, log R, and log(1/R) transformations. Although the spectral shapes remain smooth, there are still no clearly distinguishable peaks or valleys. These transformations are designed to compress high reflectance values or balance the distribution of reflectance intensities, thereby enhancing blended spectral features. However, they do not effectively amplify small differences between adjacent wavelengths, limiting their ability to highlight features relevant to tannin concentration.In contrast, **Figures 4e-h** demonstrate the effect of the first derivative transformation, which significantly improves spectral sensitivity to tannins. This is achieved by computing the rate of change between adjacent wavelengths, resulting in sharper spectral variation and an increased number of peaks and valleys. The first derivative effectively emphasizes local variations, inflection points, and abrupt changes, while suppressing low-frequency noise and reducing the impact of spectral overlap. However, this method is also more susceptible to high-frequency noise, which may reduce model robustness (Li et al., 2024; Liu et al., 2024).**Figures 4i-l** illustrate the spectral changes after applying the second derivative transformation. Compared to the first derivative, the number of spectral peaks and valleys is further increased. This occurs because the second derivative reflects the curvature rate of change in the spectral profile, which smooths flatter regions and removes weaker spectral information, thereby retaining only the most prominent features. While this transformation enhances key features, it also amplifies noise and may cause loss of useful bands, leading to reduced model stability (Wang et al., 2023; Zhong et al., 2023). Overall, although both derivative methods improve feature extraction, excessive noise sensitivity in the second derivative makes the first derivative more suitable for further modeling.

### 3.3 Extraction of characteristic wavelengths

**Figure 5:**
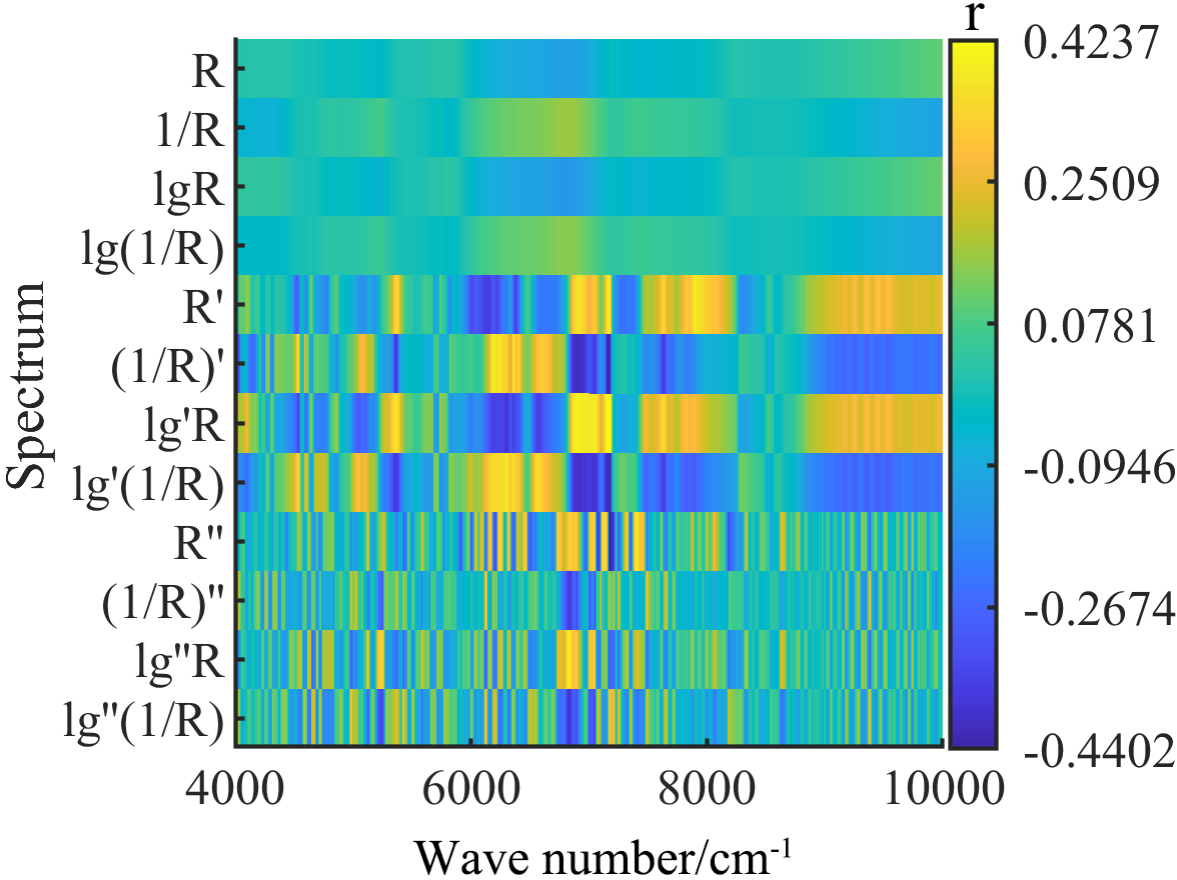
Correlation between Spectral Characteristic Bands and Tannin Content in Walnut Kernel.

The large number of wavelength variables in the original spectral data may introduce redundancy, which can impair modeling efficiency and increase computational load during subsequent analysis. Therefore, effective selection of characteristic wavelengths is essential for building efficient and accurate predictive models.Pearson correlation analysis was performed using MATLAB R2023a to assess the relationships between the measured tannin content and spectral reflectance values from both the raw spectra (R) and its 11 mathematically transformed forms. Spectral variables that passed a significance threshold of *P* < 0.01 and had correlation coefficients exceeding the critical value (|*r*| > 0.123) were retained as characteristic wavelengths.As shown in **Figure 5**, the correlation coefficient (*r*) reflects the strength and direction of the relationship between individual wavelengths and measured tannin content. The color gradient in the figure indicates the magnitude of correlation: darker tones represent stronger positive or negative associations, while lighter tones indicate weaker correlations.

**Table 1** summarizes the number of characteristic wavelengths identified under the original spectrum and various mathematically transformed spectra, along with the maximum, minimum, and mean values of their positive and negative Pearson correlation coefficients. Compared to the original reflectance spectrum (R), transformations such as 1/R, log R (lgR), and log(1/R) increased the number of selected wavelengths but did not yield significant improvements in correlation strength.In contrast, derivative-based preprocessing significantly enhanced the correlation between spectral variables and tannin content in walnut kernels. Specifically, under first derivative transformations, spectra such as R′, (1/R)′, lg′R, and lg′(1/R) produced a moderate number of characteristic wavelengths but showed substantially improved correlations, with maximum absolute *r* values of 0.399, 0.392, 0.424, and 0.386, respectively.Second derivative transformations also improved correlation levels, particularly for lg″(1/R) and lg″R, which reached correlation values as high as ±0.400. Taking both the number of selected wavelengths and the correlation strength into consideration, the mean absolute correlation coefficient (|*r*|) was used as the evaluation criterion. As a result, R′, lg′R, and lg′(1/R) were selected as the most effective mathematical preprocessing methods for subsequent model development.

**Table 1:** Statistics of the number of characteristic bands and correlation values.

Transformation	NFB	Positive correlation extremum	Negative correlation extremum	|r|
R	33	0.106	-0.127	0.041
1/R	155	0.162	-0.120	0.056
lgR	79	0.113	-0.145	0.048
lg(1/R)	79	0.145	-0.113	0.048
R’	981	0.399	-0.367	0.172
(1/R)’	877	0.392	-0.435	0.160
lg’R	1115	0.424	-0.386	0.193
lg’(1/R)	1115	0.386	-0.424	0.193
R’’	607	0.395	-0.440	0.120
(1/R)’’	339	0.324	-0.381	0.085
lg’’R	671	0.400	-0.348	0.117
lg’’(1/R)	671	0.348	-0.400	0.117

**Figure 6:**
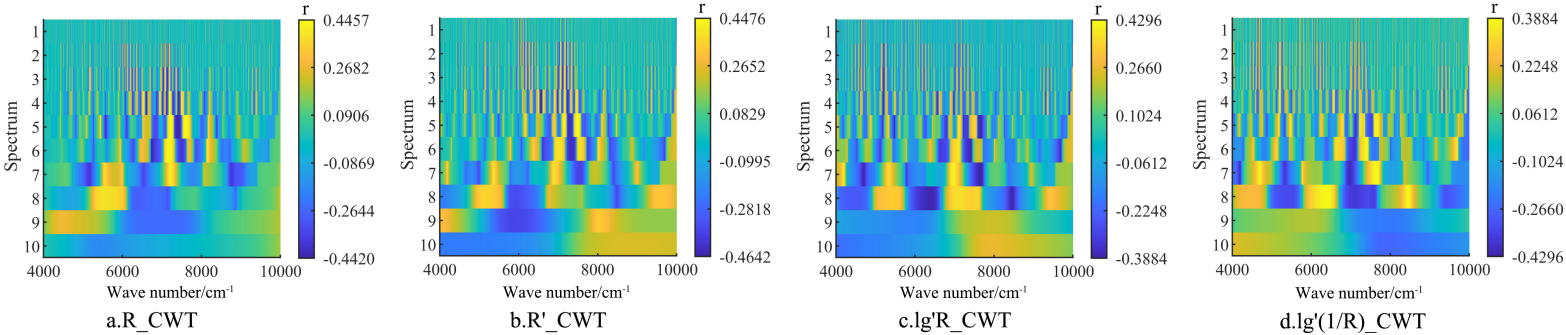
Correlation between Wavelet Coefficients and Tannins under Four Different Continuous Wavelet Transform Processing Methods. **(a)** R_CWT; **(b)** R’_CWT; **(c)** lg’R_CWT; **(d)** lg’(1/R)_CWT.

Research indicates that the wavelet transform offers significant advantages over traditional mathematical transformations in spectral processing. To investigate these advantages, continuous wavelet transform (CWT) analyses were conducted on R, R’, lg’R, and lg’(1/R) (Equations 1 and 2), denoted as R_CWT, R’_CWT, lg’R_CWT, and lg’(1/R)_CWT, respectively. R was utilized to represent the correlation between the wavelet coefficients and tannin levels in walnut kernels. As illustrated in **Figure 6**, CWT processing resulted in an overall increase in the correlation between the spectral data and walnut tannin. The correlation exhibited a trend of initially increasing and then decreasing from scale 2^1^ to 2^10^. Notably, at scale 2^4^, R_CWT and lg’R_CWT reached their maximum correlation values of 0.446 and 0.430, respectively. The maximum value for R’_CWT at scale 2^5^ was 0.448, while the maximum R for lg’(1/R)_CWT at scale 2^7^ was 0.388. As the decomposition scale increased, the number of characteristic bands across the four CWT treatments generally exhibited an upward trend (**Figure 6**).

**Figure 7:**
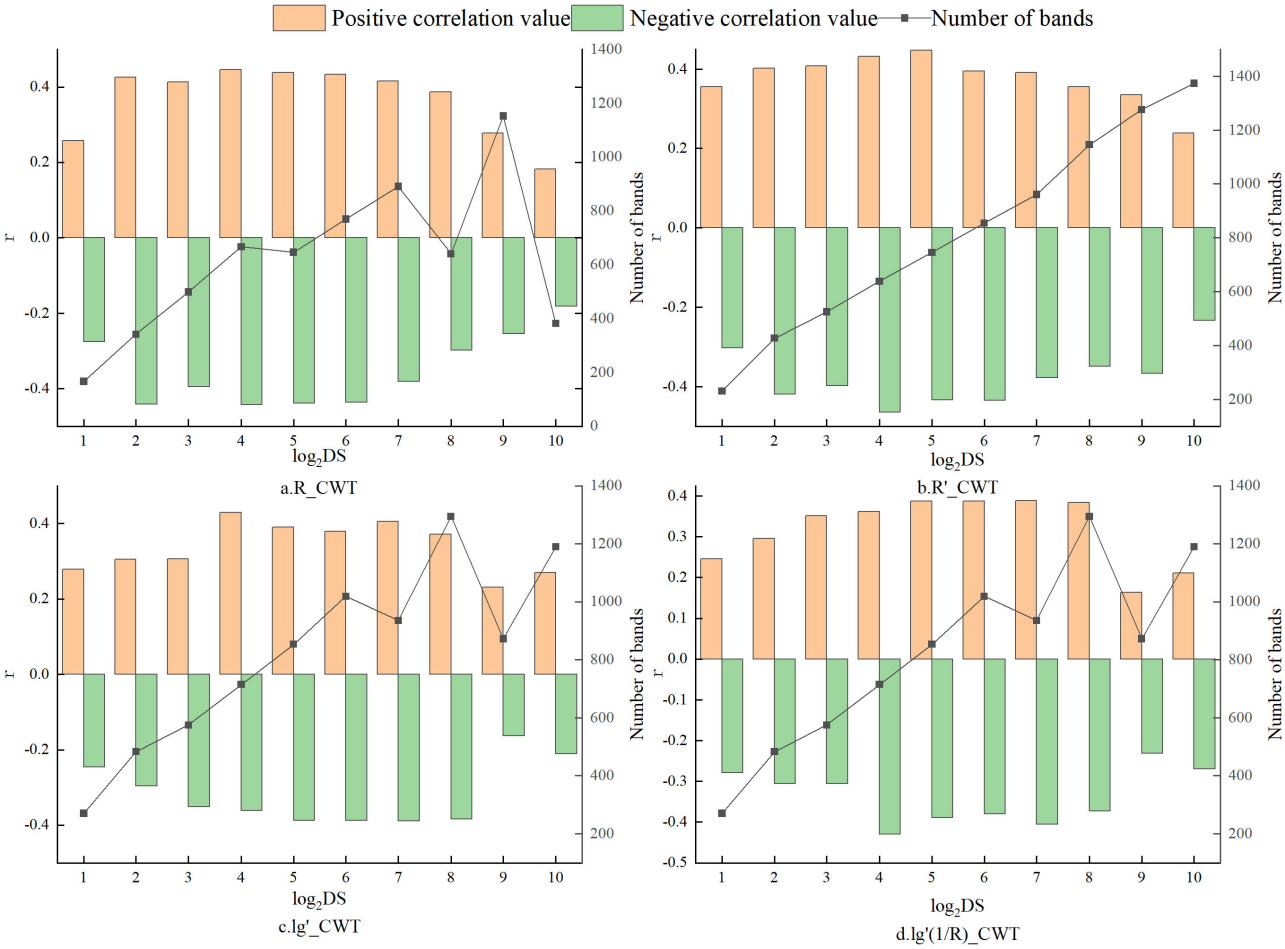
Extreme positive and negative correlations between wavelet coefficients and tannins, as well as the number of characteristic bands, under four different CWT processing methods. **(a)** R_CWT; **(b)** R’_CWT; **(c)** lg’R_CWT; **(d)** lg’(1/R)_CWT.

As shown in **Figure 7**, the mean absolute Pearson correlation coefficient (|*r*|) for R_CWT and R′_CWT reached their respective maximum values at scale 2^9^, with values of 0.178 and 0.208. For lg′R_CWT and lg′(1/R)_CWT, the highest mean |*r*| values (both 0.240) were observed at scale 2^8^.Considering both the number of characteristic wavelengths and the strength of their correlations, scale 2^9^ was selected as the optimal decomposition scale for R_CWT and R′_CWT, while scale 2^8^ was determined to be optimal for lg′R_CWT and lg′(1/R)_CWT.In subsequent tannin prediction modeling, wavelet coefficients extracted from these four spectral forms at their respective optimal scales will be used as independent variables for model construction.

### 3.4 Sample set partitioning

**Figure 8:**
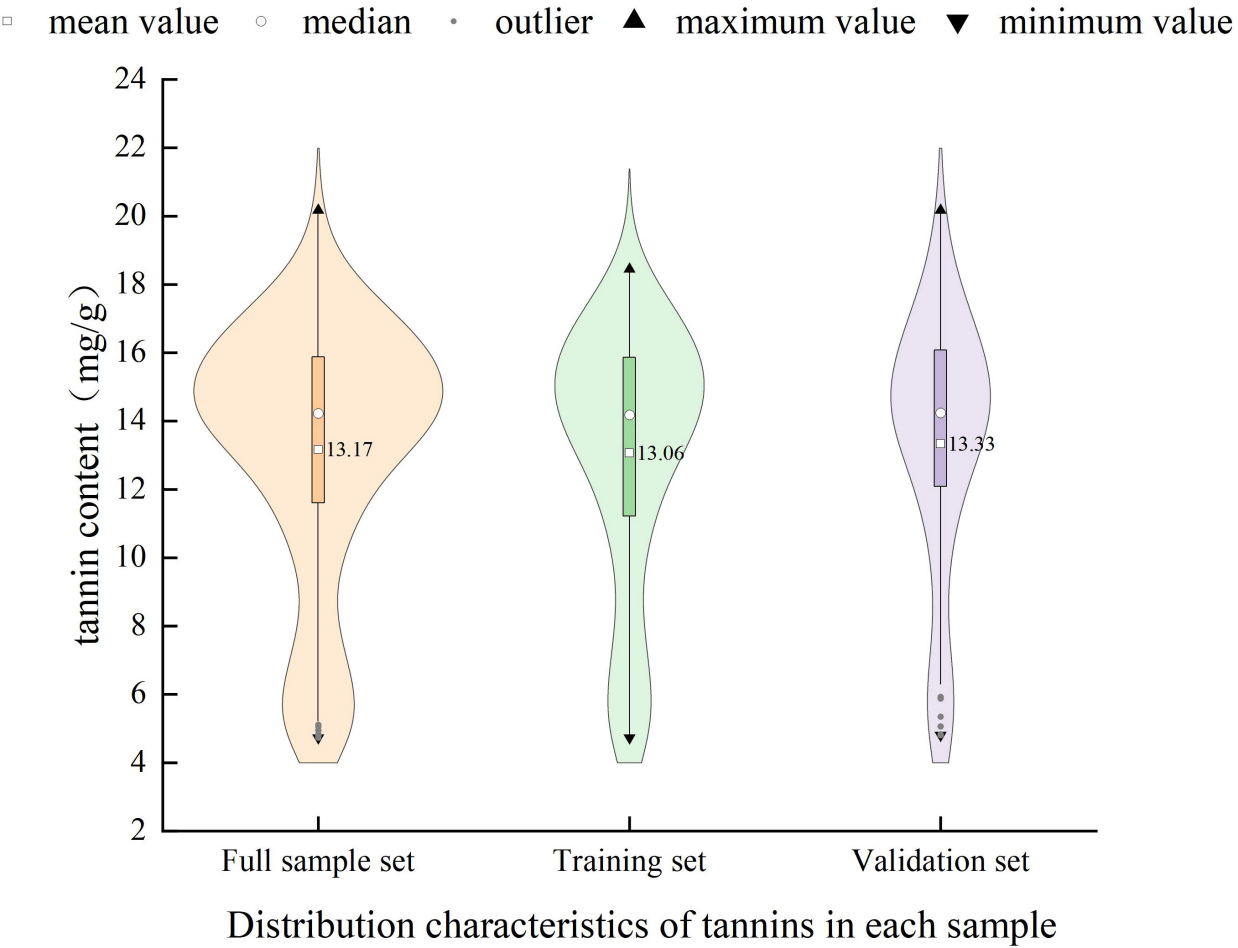
Descriptive statistical characteristics of tannins in each sample set.

Prior to model construction, the full dataset was randomly divided into training and validation sets at a ratio of 6:4. A total of 102 samples were randomly assigned to the training set for model development, while the remaining 69 samples were used as the validation set for performance evaluation. As shown in **Figure 8**, presents violin plots of tannin content for the three datasets.the tannin content across all samples ranged from 4.73 to 20.17 mg/g. The distribution patterns of tannin content were generally consistent among the full dataset, training set, and validation set. The outer contours reflect kernel density estimates, with wider sections indicating larger sample concentrations.and illustrates the distribution of standard deviation, median, and coefficient of variation (CV) across the datasets.The mean tannin content of the full dataset was 13.17 mg/g, while the training and validation sets had mean values of 13.06 mg/g and 13.33 mg/g, respectively. The mean, median, and standard deviation of tannin content were comparable across the three sets. Moreover, the full dataset exhibited CV and mean values intermediate between those of the training and validation sets, indicating that the partitioning was statistically balanced and representative. These results support the suitability of the sample division for constructing a robust and generalizable prediction model for walnut kernel tannin content.

### 3.5 Model construction and accuracy evaluation

**Figure 9:**
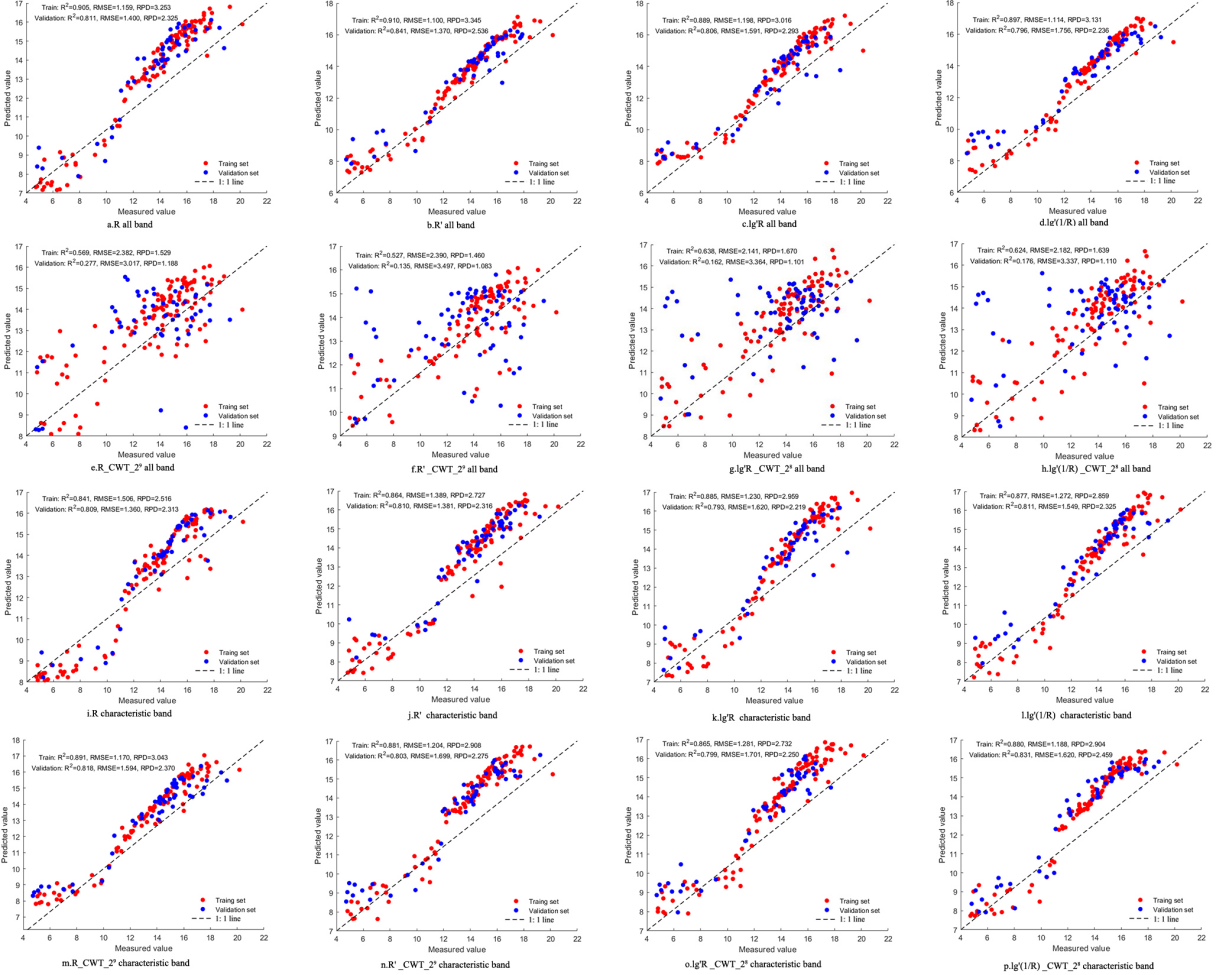
Results of Training and Validation Sets for Walnut Tannin Estimation Model. **(a)** R full band; **(b)** R’full band; **(c)** lg’R full band; **(d)** Lg’(1/R) full band; **(e)** R_CWT2^9^ full band; **(f)** R’_CWT_2^9^ full band; **(g)** lg’R_CWT_2^8^ full band; **(h)** lg’(1/R)_CWT_2^8^ full band; **(i)** R characteristic band; **(j)** R’characteristic band; **(k)** lg’R characteristic band; **(l)** lg’(1/R) characteristic band; **(m)** R_CWT2^9^ characteristic band; **(n)** R’_CWT_2^9^ characteristic band; **(o)** lg’R_CWT-2^8^ characteristic band; **(p)** lg’(1/R)_CWT_2^8^ characteristic band.

In this study, random forest (RF) models were developed to predict tannin content in walnut kernels using both full-spectrum data and selected characteristic wavelengths derived from various spectral preprocessing techniques. The spectral variables served as independent variables, while measured tannin content was used as the dependent variable.To optimize RF model performance, key hyperparameters were systematically tuned. Specifically, the number of decision trees was set to 200, the minimum leaf size was fixed at 10, Bayesian optimization was performed with 50 iterations, and five-fold cross-validation was used to ensure predictive accuracy and model generalizability. The modeling results are presented in **Figure 9**.The results showed notable differences in model performance between full-spectrum and characteristic wavelength inputs under different spectral preprocessing strategies. Overall, models based on selected characteristic wavelengths outperformed those using full-spectrum data, particularly in terms of the coefficient of determination (R^2^). Most models based on characteristic wavelengths achieved R^2^ values above 0.70, whereas nearly half of the full-spectrum models showed signs of overfitting.This indicates that selecting informative wavelengths can effectively eliminate irrelevant spectral information, thereby enhancing model performance. Moreover, under the same preprocessing method, the difference in R^2^ values between the training and validation sets was smaller when using characteristic wavelengths, further demonstrating improved model stability.Although full-spectrum models exhibited slightly better RMSE and RPD values in the training set, their RPD values differed substantially between training and validation sets—with a maximum difference of 0.928—suggesting weaker generalization capability. In contrast, the RPD values of characteristic wavelength models in the validation set were typically above 2.2, indicating higher predictive accuracy and better robustness across different preprocessing methods. Nearly half of the full-spectrum models had RPD values below 1.4, further underscoring their limited stability.In summary, feature wavelength selection reduced spectral redundancy and improved model robustness. While full-spectrum models performed slightly better in certain metrics, models based on characteristic wavelengths offered superior comprehensive performance in terms of accuracy, stability, and computational efficiency, making them more suitable for tannin estimation.Notably, models constructed using first derivative spectra combined with continuous wavelet transform (CWT) achieved the highest prediction accuracy and stability. This highlights that integrating first derivative preprocessing with CWT significantly enhances the model’s ability to predict tannin content and offers a reliable strategy for improving spectral model performance.

### 3.6 SHAP-based interpretation of the RF model

**Figure 10:**
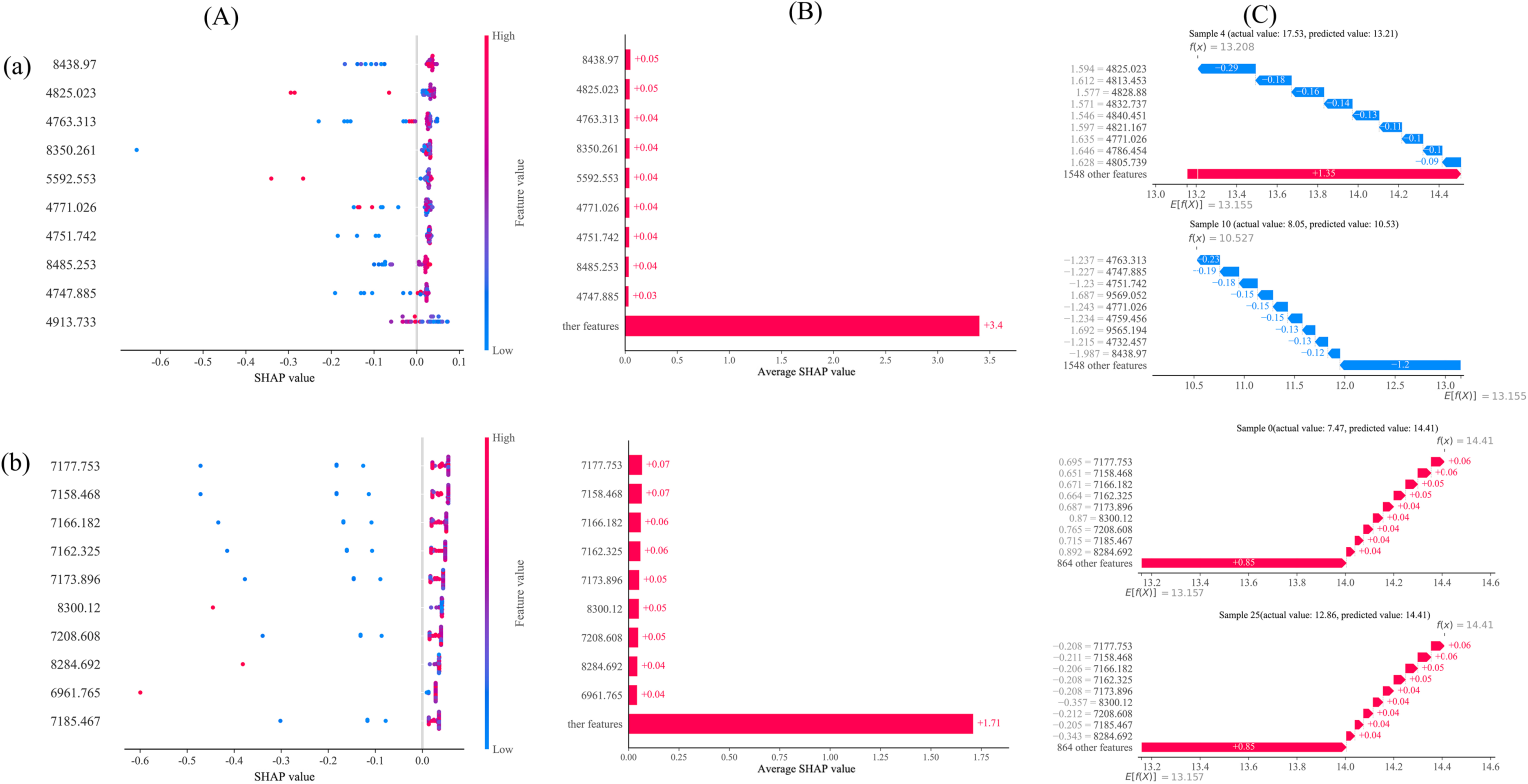
Explains the feature interpretation of the random forest model using the SHAP algorithm. **(A)** List the top 10 important features of the bee colony graph, with red data points indicating high SHAP values and blue data points indicating low SHAP values. **(B)** SHAP algorithm summary chart, representing the mean SHAP value of each feature. **(C)** SHAP algorithm waterfall diagram,randomly select 2 samples for analysis;a:lg’(1/R)_CWT_2^8^ full band;b:lg’(1/R)_CWT_2^8^ characteristic band.

Due to the inherent “black-box” nature of machine learning algorithms, assessing the influence of input features plays a crucial role in model interpretation and optimization (Ye et al., 2024). To identify the most influential spectral variables and explain their contributions to the model’s predictions, the SHAP algorithm was employed. Visualization of model interpretability was performed using the SHAP library in Python.Specifically, SHAP values were used to evaluate the contribution of each selected wavelength in the best-performing RF prediction model. As shown in **Figure 10A**, the top 10 most important features are visualized in a SHAP beeswarm plot. The features are ranked in ascending order based on their cumulative contribution to the model. It is evident that the most influential spectral regions are located within the 4000-4999 cm^-1^ and 7000-8999 cm^-1^ ranges. The horizontal axis represents SHAP values, while the vertical axis displays the features ranked by their overall impact. **Figure 10B** presents a bar plot of the mean absolute SHAP values for all features, indicating the average contribution of each variable across all predictions. Features with higher SHAP values contributed more significantly to the model’s output. **Figure 10C** shows waterfall plots for two randomly selected samples, illustrating the contribution of individual features to the prediction result. Positive SHAP values indicate an increase in the predicted tannin content, whereas negative values suggest a decrease. The vertical axis ranks the features by cumulative SHAP impact. Blue bars represent features that reduced the prediction, while red bars represent features that increased the prediction.

## 4 Discussion

This study investigated the effects of various spectral preprocessing techniques—including mathematical transformations, continuous wavelet transform (CWT), and their combinations—on enhancing spectral sensitivity and improving the predictive accuracy for tannin content. The results demonstrated that reciprocal, logarithmic, and reciprocal-logarithmic transformations did not significantly improve the correlation between spectral variables and tannin content. In contrast, the first derivative transformation markedly enhanced correlations by amplifying subtle variations within the spectral data. These findings align with previous reports by Guo Yanping et al. (2025), Li et al. (2024); Zhang et al. (2023), confirming the efficacy of first derivative processing in spectral pretreatment.

However, compared to wavelet-based methods, conventional mathematical transformations were less effective in suppressing high-frequency noise and managing complex background interference. CWT demonstrated distinct advantages in dimensionality reduction, noise suppression, and feature enhancement. Decomposing the original spectra via CWT led to notable improvements in both model sensitivity and stability, outperforming traditional mathematical preprocessing. This observation is consistent with the work of Hu et al. (2025), who demonstrated that CWT effectively extracted spectral features related to rice leaf SPAD values using a BPNN model based on bior3.3 wavelets. Similarly, Wang et al. (2022) reported that small-scale CWT significantly improved the estimation of nitrogen content in tea leaves, reducing the number of input variables by 99.34% and increasing model accuracy by 11% compared to conventional preprocessing methods.

The characteristic wavelengths identified in this study, notably those within the 4000-5000 cm^-1^ and 7000-9000 cm^-1^ ranges, correspond to known NIR absorption regions for phenolic compounds. The former region is often associated with combination bands involving O-H and C-O vibrations, while the latter is typically linked to the first overtone of O-H stretching. These assignments are consistent with the chemical structure of tannins, which are rich in hydroxyl and aromatic moieties. The strong correlation between these spectral features and tannin content underscores the physicochemical plausibility of the selected wavelengths and supports the robustness of the developed prediction model.

Overall, the integration of wavelet decomposition, particularly CWT, enhanced both model accuracy and robustness. Among the preprocessing strategies, the combination of the first derivative and CWT proved especially effective. This synergy likely stems from the first derivative’s capacity to capture fine-scale spectral variations, which are subsequently refined and enhanced through the multi-resolution decomposition provided by CWT (Yumiti· and Wang, 2022). Consequently, this combined approach yielded superior predictive performance compared to standalone mathematical or wavelet transformations. Furthermore, CWT exhibited high computational efficiency and sensitivity in detecting abrupt changes and localized features within high-dimensional spectral data. By preserving both low- and high-frequency information, CWT contributed to improved modeling performance relative to conventional spectral transformation algorithms, as also evidenced in prior studies (Yumiti· and Wang, 2022; Wang et al., 2014).In the data preprocessing stage, outlier detection and feature selection are based on all samples, and the training set and verification set are not strictly distinguished. Although this practice is common in spectral studies of limited samples (Jensen et al., 2008; Ying et al., 2006), it may introduce a certain risk of information leakage in theory, resulting in optimistic model verification results. Future research can adopt more rigorous nested cross validation or completely independent validation set design to further improve the generalization ability and reliability of the model.

While the random forest (RF) algorithm was selected for this study due to its established robustness, interpretability, and strong performance in similar spectral applications, we acknowledge that other machine learning approaches—such as gradient boosting, support vector machines, and deep learning architectures—may offer distinct advantages for spectral modeling. Future comparative studies that systematically incorporate a broader range of algorithms could further refine prediction accuracy for tannin content and provide deeper insights for model selection in this field.

In summary, CWT outperformed traditional mathematical transformations in strengthening the correlation between spectral data and tannin content and in enhancing model accuracy. Moreover, combining mathematical transformations with wavelet processing optimized the spectral pretreatment pipeline, leading to improved predictive performance. The application of diverse preprocessing methods for NIR-based tannin estimation establishes a solid foundation for monitoring walnut tannins and opens new avenues for remote sensing-based quantitative trait analysis in agriculture. Future research could integrate optimized spectral transformation techniques with advanced machine learning algorithms and satellite remote sensing data to enable regional-scale monitoring of walnut tannin content, thereby advancing the application of remote sensing technologies in fruit crop research.

## 5 Conclusion

This study used ‘Wen 185’ walnut kernels as the research material and applied multiple preprocessing strategies—including mathematical transformations, continuous wavelet transform (CWT), and their combination—to enhance spectral data. Based on these preprocessing results, random forest (RF) models were constructed to quantitatively predict tannin content. The first derivative transformation, CWT, and the combination of first derivative with CWT all improved the correlation between spectral data and measured tannin content. Among them, the combination of first derivative and CWT yielded the best model performance.The most effective prediction model was constructed using the characteristic wavelengths of lg′(1/R)_CWT at scale 2^8^, achieving R² values of 0.880 and 0.831 for the training and validation sets, respectively; RMSE values of 1.188 and 1.620; and RPD values of 2.904 and 2.459. These results indicate strong predictive accuracy and robustness.Furthermore, the SHAP algorithm was employed to visualize feature importance and model interpretability. The analysis confirmed that the RF model effectively captured the key wavelengths contributing to tannin prediction, offering a reliable, interpretable approach for estimating tannin content in walnuts.

## Data Availability

The original contributions presented in the study are included in the article/supplementary material. Further inquiries can be directed to the corresponding author.
