# Correction: Near-infrared prediction of tannin content in walnut kernels using wavelet transform combined with interpretable machine learning models

**DOI:** 10.3389/fpls.2026.1806058

**Published:** 2026-02-27

**Authors:** Qiuhao Xia, Langqin Luo, Yerhazi Yerzati, Mian Muhammad Ahmed, Yonghao Chen, Shiwei Wang, Jiangnan Qin, Liping Chen, Qiang Jin, Zhongzhong Guo, Rui Zhang

**Affiliations:** 1 College of Horticulture and Forestry, Tarim University, Alar, China; 2 Efficient and High-Quality Cultivation and Deep Processing Technology of Characteristic Fruit Trees in Southern Xinjiang, National Local Joint Engineering Laboratory, Alar, China; 3 Xinjiang Production and Construction Corps, Southern Xinjiang Characteristic Forest and Fruit Technology Innovation Center, Alar, China; 4 College of Life Science and Technology, Tarim University, Alar, China; 5 Beijing Academy of Agricultural and Forestry Sciences Forestry Fruit Tree Research Institute, Beijing, China; 6 School of Forestry and Landscape Architecture, Xinjiang Agricultural University, Urumqi, China; 7 School of Information Engineering, Tarim University, Aral, China

**Keywords:** continuous wavelet transform (CWT), near-infrared, random forest (RF), Shapley additive explanations (SHAP), tannins

There is an error in the correspondence section of the article as published. Author Zhongzhong Guo was not included as a corresponding author.

The original article has been updated.

